# Long-term *in vitro* evolution of human-associated bifidobacteria provides insights into genome-based strain delineation

**DOI:** 10.20517/mrr.2026.37

**Published:** 2026-09-03

**Authors:** Gabriele Andrea Lugli, Chiara Argentini, Giulia Longhi, Leonardo Mancabelli, Christian Milani, Francesca Turroni, Douwe van Sinderen, Marco Ventura

**Affiliations:** ^1^Laboratory of Probiogenomics, Department of Chemistry, Life Sciences, and Environmental Sustainability, University of Parma, Parma 43124, Italy.; ^2^Microbiome Research Hub, University of Parma, Parma 43124, Italy.; ^3^Department of Medicine and Surgery, University of Parma, Parma 43124, Italy.; ^4^APC Microbiome Institute and School of Microbiology, Bioscience Institute, National University of Ireland, Cork T12 Y337, Ireland.

Bifidobacteria are widely regarded as beneficial members of the human gut microbiota and play a central role in mother-to-infant vertical transmission, as well as in the maintenance of intestinal homeostasis throughout life^[1]^. Among the human-associated species, *Bifidobacterium bifidum*, *Bifidobacterium breve*, and *Bifidobacterium longum* are particularly relevant during early life, whereas *B. longum*, together with *Bifidobacterium adolescentis*, remains prominent in later life stages through adolescence, adulthood, and into old age^[2,3]^. Although many studies have focused on identifying strain transmission events, it is still unclear whether a robust molecular clock can be defined to trace the evolution of bifidobacterial strains over time^[4]^.

In this context, controlled *in vitro* evolution experiments are essential to estimate the number of small genomic variants that can accumulate within a defined time window and, therefore, to provide a framework that highlights strain diversification. Such experiments help disentangle true evolutionary changes from background technical noise and provide the basis for evaluating whether thresholds based on single nucleotide polymorphisms (SNPs) or short insertions/deletions (INDELs) are suitable to infer strain continuity or divergence. This issue is particularly relevant since thresholds are increasingly used to monitor strain transmission in population studies, where whole-genome sequence data are used to determine whether two isolates can still be considered clones of the same original strain within a given timescale^[5,6]^.

However, interpretation of genomic distances depends heavily on the availability of an adequate genomic dataset. Reliable strain-level inference requires an exhaustive and well-curated genome database, ideally capturing the overall genetic diversity within the species under investigation. At the same time, genome dereplication remains necessary to avoid overrepresentation of nearly identical genomes that may artificially bias downstream analyses of gut microbiota. This point is particularly important for metagenomic applications, where average nucleotide identity (ANI)-based clustering and database dereplication are common practices. By contrast, when only a handful of complete genomes are available, dereplication becomes less informative, and the limited genomic diversity represented in the reference database may reduce the resolution of strain-tracking approaches.

From an applied perspective, strain identity has different implications depending on the field of application, including strain assignment, strain tracking, and industrial release of microbial products. A 95% ANI threshold is informative for species delineation, while probiotic manufacturing and batch-to-batch quality control require discrimination among nearly identical genome sequences belonging to the same deposited strain. For this reason, a very high ANI range, interpreted together with small genomic variant counts, may be more useful for verifying batch continuity, protecting strain ownership, and identifying unwanted laboratory domestication. We therefore discuss our data as a practical benchmark for pure cultures under controlled propagation conditions, which should be complemented by additional product-specific phenotypic assays to validate strain performance and production quality. This is particularly important for protecting the rights of the strain’s owner against fraudulent practices, such as isolating the bacterium from a probiotic product and subsequently renaming it under a different strain designation.

Here, we report data from a pilot study involving four bifidobacterial strains belonging to the most relevant human-associated species, namely *B. bifidum* PRL2010, *B. breve* PRL2012, *B. longum* PRL2022, and *B. adolescentis* PRL2023^[2,3]^, subjected to longitudinal screening to monitor acquisition of small genomic variants across their genomes. Each strain was propagated as a single continuous lineage, without the establishment of parallel independent evolutionary lineages. To investigate species-specific evolutionary trajectories, the four strains were individually grown in 10 ml of MRS medium (Scharlab Chemie, Spain) supplemented with 0.05% (wt/vol) L-cysteine hydrochloride (Merck, Germany) and incubated at 37 °C in a Concept 400 anaerobic chamber (Baker, Ireland) under an atmosphere composed of 2.99% H_2_, 17.01% CO_2_, and 80% N_2_. Each day, 100 µL of culture was transferred into 9.9 ml of fresh MRS medium, corresponding to a 1:100 dilution. Over 18 months, this daily procedure comprised 546 passages, equivalent to approximately 6.6 generations per passage [log_2_(100)] and roughly 3,600 generations per lineage. This serial-propagation scheme provided stable cultivation conditions for the gradual emergence and maintenance of spontaneous variants during the longitudinal experiment.

Strain identity was systematically monitored by a MALDI-TOF biotyper (Bruker, USA) to confirm culture integrity prior to reinoculation, while 16S rRNA gene amplicon sequencing was performed every three months to verify the absence of contamination. Frozen glycerol stocks were prepared at each whole-genome sequencing (WGS) sampling point, and DNA for WGS was extracted directly from samples of the evolving bulk culture, rather than from single-colony isolates. Whole-genome sequencing was carried out at four time points, namely at the start of the experiment (T0), after six months (T1), 12 months (T2), and 18 months (T3) [Figure 1A], using an Illumina NextSeq 2000 platform (Illumina, USA) with a 300-cycles reagent kit, generating an average of 2.9 ± 0.8 million reads per sample [Supplementary Table 1]. Altogether, this workflow yielded a high-quality genome dataset, corresponding to an average genome coverage of 353 ± 94.

**Figure 1:**
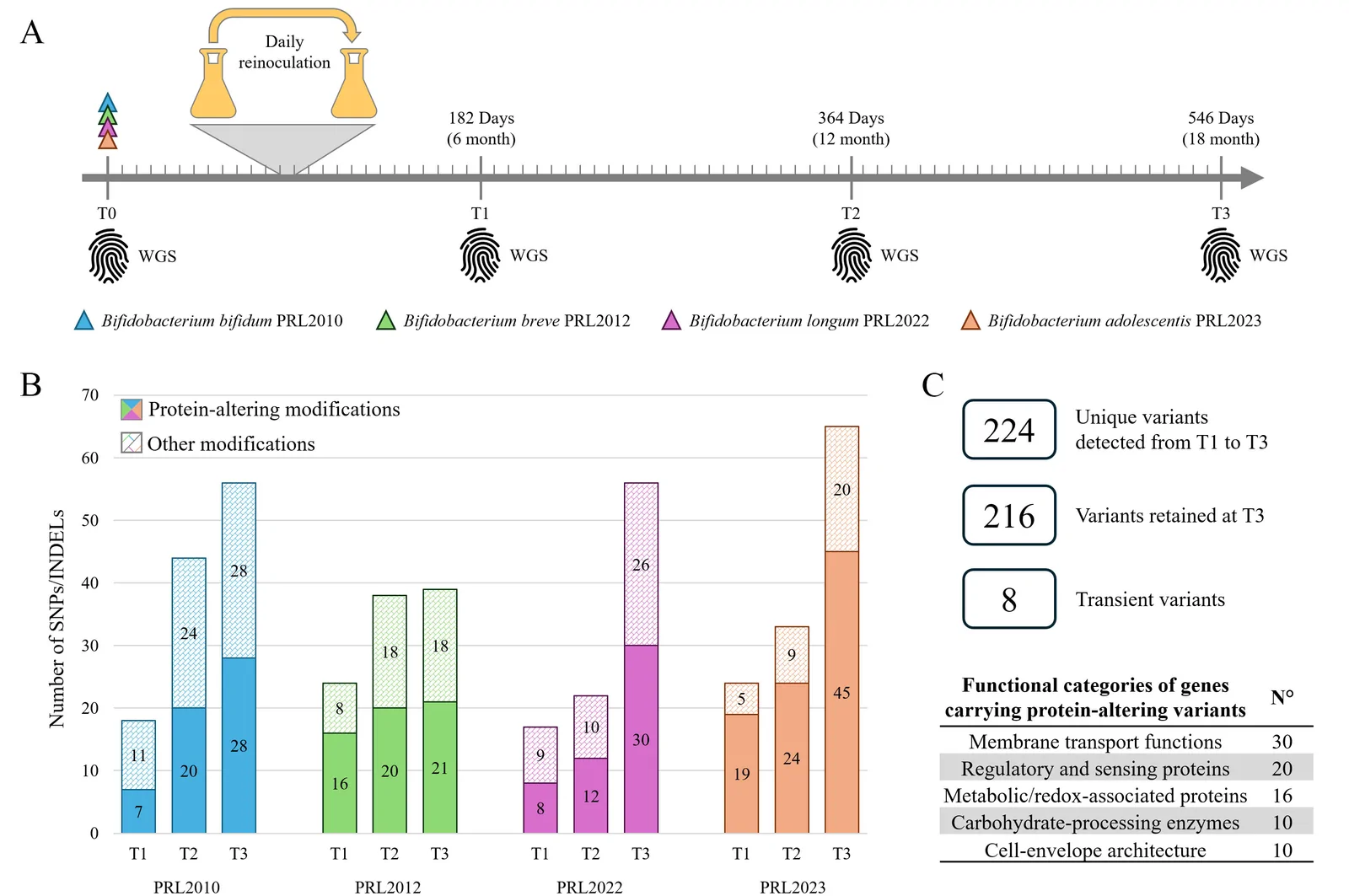
Experimental design and longitudinal accumulation of genetic variation in four bifidobacterial strains during prolonged *in vitro* cultivation. (A) depicts the experimental workflow, including four human-associated bifidobacterial strains maintained as single continuous lineages, serially reinoculated daily at a 1:100 dilution. Whole-genome sequencing (WGS) was performed at T0, T1, T2, and T3, using T0 data as a reference for variant calling; (B) shows a stacked bar plot with the progressive accumulation of SNP/INDEL variants detected at each sampling point in each strain. The filled portion of each bar indicates the number of protein-altering variants affecting the amino acid sequence of translated genes, whereas the hatched portion represents the remaining detected variants; (C) reports the total number of unique variants detected during the study, variants retained at T3, and transient variants, and summarizes the main predicted functional categories affected by protein-altering variants. T0: The start of the experiment; T1: six months; T2: 12 months; T3: 18 months; SNP: single nucleotide polymorphism; INDEL: insertion/deletion.

Small genomic variants were defined as SNPs and short INDELs detected relative to the corresponding T0 reference genome. Reads were aligned with BWA-MEM2^[7]^ and the resulting alignments were sorted and indexed with SAMtools^[8]^. Variant calling was performed with FreeBayes in haploid pooled-continuous mode, using a minimum coverage of 10 reads, a minimum alternate-allele count of 2, and a minimum alternate-allele fraction of 0.01. For downstream reporting, only SNPs and INDELs supported by at least five alternate reads were retained, and high-confidence variants were selected using an alternate-allele fraction threshold of 70%. To minimize mapping artifacts and assembly-related biases, we first identified self-variants by remapping the sequencing data originally used to assemble the complete reference genome of each strain (T0). This strategy allowed us to filter out *in silico* background discrepancies attributable to hybrid genome assembly and to compare only genuine sequence changes that emerged at each time point. Variant effects were marked using gene annotations produced by MEGAnnotator2^[9]^ from T0 genomes, grouping variants into synonymous, nonsynonymous, frameshift, stop-gained, and non-coding/intergenic categories. Nonsynonymous substitutions were classified as protein-altering candidate variants because they modify the predicted amino acid sequence, whereas frameshift and stop-gained variants were treated as putative high-impact coding events whose actual functional consequences require phenotypic validation. Furthermore, genome assemblies were performed by MEGAnnotator2^[9]^ [Supplementary Table 1], and genomic divergence accumulated during the 18-month experiment was quantified using four ANI-based approaches, *i.e.*, skani, pyANI, ANIb, and fastANI^[10-12]^.

The analysis identified 224 unique small variants across the four strains over the 18-month experiment. This count represents all distinct SNP/INDEL events detected at least once from T1 through T3, with strain-level totals ranging from 42 unique variants in *B. breve* PRL2012 to 70 in *B. adolescentis* PRL2023 [Figure 1B]. When variants were counted at each sampling point, 83 were present at T1, 137 at T2, and 216 at T3. Thus, 216 variants were retained after 18 months, whereas eight variants detected earlier were transient. The number of variants at T3 was 56 for *B. bifidum* PRL2010, 39 for *B. breve* PRL2012, 56 for *B. longum* PRL2022, and 65 for *B. adolescentis* PRL2023, indicating that most variants, once detected, were subsequently maintained. The proportion of transient variants ranged from 0% in *B. bifidum* PRL2010 to 7.7% in *B. adolescentis* PRL2023. With respect to their predicted function, 123 genes were affected by at least one variant. Among them, 105 variants produced nonsynonymous substitutions, thereby altering the predicted encoded amino acid sequence, while 23 additional events were classified as higher-impact coding events, including frameshift-causing INDELs and SNP-derived stop codons [Figure 1B].

While protein-altering variants occurred across the entire genome, the affected genes predominantly belonged to a restricted set of biologically meaningful functional categories [Figure 1C and Supplementary Table 2]. The largest group comprised membrane transport functions (30 genes), such as MFS transporters, ABC transporters, PTS system components, and antiporters, indicating selective pressure on nutrient uptake and membrane homeostasis. The second most recurrent targets were regulatory and sensing proteins (20 genes), including transcriptional regulators and sensor kinases, suggesting adaptive rewiring of environmental response pathways. Variants also affected 10 carbohydrate-processing enzymes and 16 central metabolic/redox-associated proteins involved in sugar utilization, glycolysis, and related metabolic functions, consistent with metabolic remodeling during prolonged cultivation. Finally, 10 genes were linked to cell-envelope architecture, cell division, and surface-associated functions, suggesting possible changes in cell physiology and interaction with the surrounding environment. These predictions do not demonstrate altered industrial performance on their own, but they identify testable hypotheses for future assays of growth kinetics, acidification capacity, stress tolerance, cell-surface traits, and the production of strain-specific nutritional metabolites.

Taken together, these results indicate that after 18 months of serial growth in MRS medium, the four bifidobacterial strains exhibited a consistent pattern of *in vitro* adaptation to a stable and nutrient-rich environment. More specifically, the observed variants suggest progressive adaptation to the recurrent physiological cycles imposed by serial propagation in MRS, including the lag, exponential, stationary, and decline phases. In this context, variants affecting sugar uptake and transport (including PTS, ABC, and MFS systems), carbohydrate processing (such as beta-galactosidase and glycoside hydrolase functions), and central carbon metabolism (including glucokinase, glyceraldehyde-3-phosphate dehydrogenase, and pyruvate kinase) are consistent with optimization of nutrient acquisition, metabolic flux, and tolerance to stresses arising during late growth phases, including medium acidification [Supplementary Table 2]. The recurrent detection of frameshift and stop-gained variants further supports the notion of laboratory domestication, whereby functions that are less advantageous under highly predictable culture conditions may become dispensable and therefore accumulate loss-of-function alterations. Notably, we perform the experiment in 18 months as an empirical genomic upper boundary for laboratory-maintained bifidobacterial pure cultures under comparable conditions, rather than as a validation threshold for industrial functionality or shelf life. Indeed, industrial microbial starters are generally not subjected to such prolonged serial reinoculation over many months. Therefore, under industrial settings, our genomic framework should be integrated with phenotype-based assays, performance evaluations, and viability assessments after production and storage.

Although the experimental setting and sampling intervals differ substantially from those encountered *in vivo*, our findings are broadly consistent with previous observations reporting a mean of 22.79 SNPs (range 3-61) among vertically transmitted bifidobacterial strains recovered from 24 mother-infant dyads during the first and third months postpartum^[4]^. Clearly, the selective pressures acting on bifidobacteria within the infant gut are profoundly different from those imposed by prolonged laboratory cultivation. Therefore, our data should not be interpreted as a direct model of *in vivo* bifidobacterial evolution. Instead, they are more appropriately viewed as a framework for understanding strain diversification in laboratory-maintained or industrially grown settings.

Another widely used approach to estimate genomic relatedness among microorganisms is the comparison of ANI genome values, which has been applied for more than a decade to compare whole chromosomal sequences^[13]^. ANI is commonly used for species delineation, typically adopting an arbitrary threshold of 95%. At the same time, ANI can also capture genomic divergence among strains within the same species and may therefore be informative for identifying fine-scale diversification, such as the emergence of subspecies. In addition, ANI-based tools are routinely used to dereplicate genome collections by collapsing highly similar strains prior to pangenome analysis^[14]^.

Using the genome sequences obtained from T0 to T3, we compared four ANI-based algorithms, namely skani, pyANI, ANIb, and fastANI, to assess the extent of genomic divergence accumulated over the 18-month experiment^[10-12]^. Across all comparisons, skani and pyANI consistently returned values of 99.99% to 100%, while ANIb, a BLASTN-based fragment alignment approach, yielded 100% in all cases [Supplementary Table 3]. By contrast, fastANI produced a slightly broader range, with values spanning from 99.96% to 99.99%, including 99.96% for *B. longum *PRL2022, 99.98% for *B. adolescentis* PRL2023, and 99.99% for *B. bifidum* PRL2010 and *B. breve* PRL2012. This difference is expected, as fastANI estimates ANI using reciprocal mappings of 3-kb orthologous fragments and is slightly query/reference-asymmetric, whereas skani relies on symmetric approximate mapping with aligned fraction, sourmash-based pyANI tends to compress extremely similar genomes toward 100%, and ANIb is based on BLASTN-derived local alignments of genome fragments^[10,11]^. Taken together, these results indicate that genomes of the same strain consistently show ANI values above 99.99% for skani, pyANI, and ANIb, while fastANI values should be reported separately and interpreted as method-dependent variation rather than evidence of meaningful strain divergence [Supplementary Table 3].

In metagenomic studies, ANI thresholds of 99% or 99.9% are often used to cluster or dereplicate genomes at the strain level. However, the most appropriate threshold should always be interpreted in light of the specific algorithm, genome quality, genome completeness, and alignment strategy used to estimate ANI. In the present study, in which near-complete genome sequences from pure isolates were compared, our data support a more stringent value of approximately 99.99% as a practical pure-isolate benchmark for strain continuity. This estimate is consistent with recent studies that have defined a global ANI cutoff for strain-level diversity^[12,15,16]^ and have also proposed a broader ANI interval of approximately 99.5 to 99.8% to delineate genomovars, which are expected to display substantial genomic remodeling and, consequently, phenotypic differences. By contrast, our longitudinal data indicate that time-separated derivatives of the same strain remain above this interval and cluster more consistently around 99.99%, with most ANI methods, a range in which phenotypic divergence would not be expected to arise. However, we avoided proposing this value as a universal species-wide or cross-taxon threshold. Our estimate is based only on controlled pure-culture experiments involving strains cultivated in isolation, with comparable sequencing quality, and processed by four specific ANI algorithms. By contrast, strains residing in complex microbial communities such as the gut microbiota may accumulate additional genomic differences through horizontal gene transfer mediated by phages, plasmids, and other mobile genetic elements.

Overall, the variant data and genomic analyses indicate that bifidobacterial strains maintained under stable laboratory conditions accumulate a limited but measurable number of genomic changes, while still preserving an almost complete genome-wide nucleotide identity. These observations also have broader implications for metagenomic strain-tracking methods, including recently developed tools such as TRACS, which infer strain-level transmission from population-scale sequencing data^[17]^. More generally, the information reported here may prove useful in applied contexts that require stringent strain authentication, including the monitoring of probiotic strains used in industrial production and commercial formulations. In such settings, combining SNP/INDEL-based thresholds with very high ANI cutoffs may provide a practical framework to verify strain legitimacy, detect unwanted diversification during microbial growth, and support more robust quality-control pipelines. From our perspective, the next objective in industrial production should be to move from purely genomic strain identity toward a paired genotype-phenotype definition of stability. Future probiotic quality-control assays should therefore integrate longitudinal WGS, standardized ANI screening, and phenotypic assays that measure growth kinetics, survival during formulation, acidification, metabolite production, and persistence in host-relevant models. Such an integrated framework would transform strain delineation from a static nomenclatural exercise into a predictive tool for managing industrial cultures and interpreting strain transmission in human microbiome studies.

## DECLARATIONS

### Availability of data and materials

Raw genome sequences can be accessed via the SRA study PRJNA1477938.

### Copyright

© The Author(s) 2026.
